# Perceived comfort with weight, body shape and eating pattern of young adults with type 1 diabetes and associations with clinical and psychological parameters in a clinical setting

**DOI:** 10.1186/s40337-024-01059-z

**Published:** 2024-07-30

**Authors:** Sneha Vidyasagar, Alison Griffin, Helen d’Emden, Christel Hendrieckx, Neisha D’Silva

**Affiliations:** 1 Queensland Diabetes and Endocrine Centre, Mater Hospital, Brisbane, Cnr Raymond Terrace, South Brisbane, QLD 4101 Australia; 2 QIMR Berghofer Medical Research Institute, Brisbane, Australia; 3 Diabetes Australia, Brisbane, Australia; 4 Australian Centre for Behavioural Research in Diabetes, Diabetes Victoria, Carlton, VIC Australia; 5 School of Psychology, Deakin University, Geelong, Australia; 6 Institute for Health Transformation, Deakin University, Burwood, Australia; 7 University of Queensland, Brisbane, Australia

**Keywords:** Young adult, Type 1 diabetes, Disordered eating, Diabetes psychosocial assessment tool, Body satisfaction

## Abstract

**Background:**

Higher prevalence of disordered eating in young adults with type 1 diabetes (T1D) culminates in higher levels of morbidity and mortality. In addition to validated questionnaires for diabetes distress, depression/anxiety symptoms and emotional well-being, the Diabetes Psychosocial Assessment Tool (DPAT) includes three questions about comfort with weight, body shape and eating pattern (WSE), which were derived from literature and multidisciplinary team consensus. Recognising individuals with low comfort with WSE, is the first step towards identifying those who may be at risk of developing eating disorders.

**Aims:**

Observe comfort with WSE, in young adults with T1D, and its associations with demographic/clinical characteristics and psychological parameters.

**Methods:**

276 young adults, aged 15–26, who attended routine clinical care at a Young Adult Diabetes Clinic, completed the DPAT. The WSE questions were scored on a 5-point Likert scale (1 indicating lowest comfort). Linear regression analysed differences in comfort with weight and eating pattern by demographic and psychological parameters.

**Results:**

1 in 3 young adults (29%) reported low comfort with WSE (scores 1 or 2). In females, 40%, 41% and 35% had low comfort with weight, shape and eating patterns respectively, in comparison to males in whom it was 18.5%, 16% and 21.5%. Females reported lower comfort with weight and eating pattern (mean 2.9 and 3.0 respectively) than Males (mean 3.7 and 3.6 respectively), each *p* < 0.001. Lower comfort with weight (*p* < 0.001) and eating pattern (*p* = 0.001) was associated with higher body mass index (BMI). Young adults with low comfort with weight and eating pattern experienced elevated diabetes distress and depressive/anxiety symptoms (each *p* < 0.001), also when adjusted for sex and BMI.

**Conclusions:**

The study has shown that low comfort with WSE is common among young adults with T1D. Adding these questions into routine care, can allow for easy and early identification of low comfort, initiation of a therapeutic dialogue and implementation of focused management strategies.

## Abstract (plain language summary)

There is a higher rate of disordered eating (DE) in young adults with type 1 diabetes (T1D) compared to their peers without diabetes. DE occurs on a spectrum from mildly distorted thoughts and behaviours regarding weight, shape and eating to thoughts and behaviours with medical and mental health consequences that do not meet formal diagnostic criteria for an eating disorder. Early screening for DE in T1D is not routinely performed and therefore often remains undetected. Due to the seriousness of the conditions, identification is key. The Diabetes Psychosocial Assessment Tool was developed to annually assess psychosocial well-being of young adults (≥ 16 years) with T1D and includes three questions about comfort with weight, shape and eating pattern (WSE). 1 in 3 young adults with T1D reported low comfort with WSE. Lower comfort with weight and eating pattern was associated with females and with higher body mass index. There was a strong relationship between low comfort with weight and eating pattern and diabetes distress and depressive/anxiety symptoms, after adjusting for sex and body mass index. Hence, inclusion of the WSE questions is valuable for early identification of young adults with T1D who may be at risk for an eating disorder.

## Background

Disordered Eating (DE) often occurs during adolescence and young adulthood, with a median age at onset estimated at 14–19 years [1–3]. DE is more common in adolescents with T1D compared with peers without, with a prevalence of 39.3% in those with T1D and 32.5% in those without T1D [2]. In another study in youth and young adults (10–25 years), 21.2% of participants with T1D had features of DE [3]. In this study, the prevalence of DE among those with T1D was highest in the 15–19 age group (24.9%). In the Diabetes MILES Youth Study which assessed the impact of diabetes on the psychosocial outcomes of 477 Australian youth (13–19 years) with T1D, DE was assessed using the Diabetes Eating Problem Survey-Revised questionnaire (DEPS-R). 50% of females and 18% of males had a DEPS-R total score above the cutoff ≥ 20, indicative of an increased risk of engaging in DE [4]. This is partially due to the food-centric nature of diabetes self-management. Insulin omission/restriction is a unique, compensatory DE behaviour in people with T1D, for weight loss, as insulin deficiency results in a catabolic state [4–6].

A longitudinal study identified that of the 22% of female adolescents with T1D who reported DE at baseline and/or at 1-year follow-up, 92.3% of these girls also reported DE at later stages of the study (up to 5 years), suggesting its persistent nature [5].

Whilst DE does not meet diagnostic criteria for an ED (DSM-5 or ICD-10) it exists in a continuum and still confers harm to the individual’s health [6]. People with T1D could possibly manipulate their weight by restricting or omitting insulin and experience weight loss when underdosing insulin [1]. This behaviour causes an increased risk of diabetic retinopathy, diabetic ketoacidosis, malnutrition, menstrual abnormalities and poor bone health [1, 6, 7]. Those with established ED and T1D have increased 10-year mortality rates (35% of females with both Anorexia Nervosa (AN) and T1D compared with 6.5% with AN alone and 2.5% with T1D alone) [8].

In the general population, 55–98% of those with an ED have a concurrent mood/anxiety disorder [6]. Similarly, DE/ED in adolescents with T1D is associated with impaired psychosocial parameters including negative affect, poor relationships, anxiety and/or diabetes distress [6, 9].

National and International Guidelines recommend regular assessment of psychological well-being in those with T1D but this is often not implemented in clinical practice [6, 10–12]. In an Australian study in paediatric and young adult clinics, 7 out of 10 clinics felt that the screening methods used in their practice were inadequate in identifying disordered eating, with the primary barriers to screening including time pressures (90%), absence of a screening tool clinicians felt was appropriate to use (80%), and lack of staff knowledge (70%) [13]. The Diabetes Attitudes, Wishes and Needs (DAWN) survey, highlighted inadequate clinical awareness of psychological distress among clinicians [14]. The average time between the diagnosis of T1D and a subsequent ED diagnosis is 10 years [15]. Due to the seriousness of these two conditions, early detection and intervention for DE is key to prevent progression of DE.

Prospective studies have identified risk factors that predict onset of any ED, such as pressure for thinness, pursuit of the thin appearance ideal and body dissatisfaction [16–19]. Young people with T1D who perceive greater disturbances with body image, shape and weight were more likely to omit insulin compared to those who do not [4, 20].

Recognising this problem and acknowledging the recommendations, the Diabetes Psychosocial Assessment Tool (DPAT) was developed by Mater Hospital Brisbane, implemented by the Queensland (Statewide) Diabetes Clinical Network and available at Queensland Clinical Excellence Website (See Appendix 1) [21].

An extensive multidisciplinary team’s (endocrinologist, dietitian, diabetes nurse educator, psychologist, psychiatry, occupational therapy expertise) consultative process resulted in a 43-question psychosocial screening tool i.e. the DPAT. The development of this tool was informed by the literature and clinical experience of this multidisciplinary team (MDT). The DPAT incorporates three validated screening tools that assess diabetes distress (PAID) [22], depression and anxiety symptoms (PHQ-4) [23] and emotional well-being (WHO-5 Index) [24], along with clinically relevant questions around social support, financial concerns, fear of hypoglycaemia and hypoglycaemia awareness. People with all types of ED experience extreme concerns about weight, body shape and eating [6]. After the review of current literature, three broad questions of comfort with Weight, Body Shape and Eating pattern (WSE) were incorporated into the DPAT. The MDT felt it was not appropriate for all clinic attendees to complete a full DE/ED questionnaire (such as the DEPS-R). The WSE questions were chosen to identify weight and shape discomforts (body image concerns, a precursor to DE/ED) and disturbed eating patterns (associated with DE/ED) [25]. It was felt these questions were relevant to all attendees as a conversation starter and initiate the step-wise process of assessing and/or managing DE/ED with the aim of prevention.

The aims of this study were to examine (1) comfort with Weight, Body Shape and Eating Pattern (WSE) in young adults with T1D; (2) the associations between comfort with WSE and demographic and clinical characteristics; (3) the associations between comfort with WSE and diabetes distress, depressive/anxiety symptoms and emotional well-being assessed by the DPAT. We wished to confirm these associations within our specific cohort to ensure that our assessment and management strategies could be more precisely targeted for the young adult population using the WSE questions.

## Methods

### Setting

This study was undertaken at the Young Adult Diabetes Clinic (16–25 years) in the Mater Hospital Brisbane, a tertiary referral centre staffed by a MDT, including endocrinologists, diabetes nurse educators (DNE), and a dietitian. The centre also has a young adult support unit (psychologists and a psychiatrist).

We have previously reported the DPAT’s feasibility and acceptability with 100% uptake and completion by young adults attending clinic [21]. DPAT scores are used to direct a treatment pathway including access to psychology services or additional diabetes management support (Appendix 1). This tool has been used annually in routine clinical care at the Diabetes Service since 2016.

### Participants and procedures

In this cross-sectional study, participants were young adults, who attended appointments between November 2016 and January 2020 and who completed the DPAT. Exclusion criteria were participants with other types of diabetes (i.e. Latent autoimmune diabetes in adults, Type 2 diabetes mellitis, Maturity-onset diabetes of the young) and those who did not complete the WSE questions.

The specified age range of the Young Adult Diabetes Service is 16 to 25 years. Some young people transitioned into the service earlier (before the age of 16) or transitioned out of the service later (above the age of 25). As the analysis makes use of clinical data collected in routine care, the participants outside of the pre-set age range were included for completeness.

The paper-based DPAT form was completed individually just prior to the appointment and took approximately 10 min to complete. The clinic nurse reviewed and scored the DPAT responses prior to the endocrinologist review. The scores provided a recommended referral pathway to a Health Care Professional (HCP) such as a DNE, dietitian or psychologist, but the referral was only actioned at the discretion of the endocrinologist, following the consultation (Appendix 2).

For this analysis, only the first DPAT completion for each participant was used. DPAT forms were de-identified and responses stored in a password protected electronic file at the Mater Hospital. Ethics approval was obtained (HREC/18/MHS/110).

### Measures

Demographic and clinical data were collected from the medical records on the day of DPAT completion: type of diabetes, age of onset, insulin regime, HbA1c and BMI. Biological sex was obtained from the electronic medical records (EMR). During the years of data collection (2016–2020), the data entered into the EMR was either Male or Female.

The PAID scale consists of 20 diabetes-specific items pertaining to the burden and distress of living with and managing diabetes. Individuals graded each item on a 4-point Likert scale from “0 = not a problem” to “3 = serious problem” [26]. Item responses are summed and multiplied by 1.25. A score ≥ 40 indicates severe diabetes distress and is used for research and screening purposes [14]. To enhance the clinical utility of the DPAT for care provision, the MDT chose scores ≥ 30 as an indicator of moderate-severe diabetes distress, with referral to a DNE initiated at this time.

The Patient Health Questionnaire-4 (PHQ-4) measures symptoms of anxiety (2 items) and depression (2 items) over 2 weeks, on a 4-point Likert scale (0–3). A total score is calculated by adding the item scores, with a total score of ≥ 3 on each of the two anxiety or depression items being an indicator for further inquiry for the absence or presence of anxiety or depressive disorders [23, 27]. A PHQ-4 score ≥ 3 for questions 1 and 2 or questions 3 and 4, prompted a recommendation to refer to a psychologist or initiation of a General Practitioner mental health care plan.

The World Health Organisation- Five Well-being Index (WHO-5) is a positively phrased 5-item questionnaire assessing general emotional well-being, with each question scored between 1 and 5 [24, 28]. The total score is multiplied by 4 to give a total out of 100. Scores less than 28 are suggestive of likely depression in youth with T1D when modelled against the Centre for Epidemiologic Studies Depression Scale (CES = D)[24]. A WHO-5 score ≤ 28 prompted a recommendation to refer to a psychologist or initiation of a General Practitioner mental health care plan.

The DPAT contained the statements, ‘I am comfortable with my current weight’,’I am comfortable with my body shape’ and ‘I am comfortable with my eating pattern’. Each item is scored on a 5-point Likert scale where 1 indicates the lowest comfort. For scores of 1 or 2 for any of the WSE items, a referral to a dietitian is recommended. Based on the clinic’s experience, occasionally a young person’s concerns in these areas can be addressed simply by education around healthy eating and close follow-up. An example is if a person had legitimate concerns with weight due to an elevated BMI. However, after dietetic consultation, any clinical indication of DE/ED initiated a psychology referral.

### Statistical analysis

Data is summarised using n (%) if categorical, mean (standard deviation (SD)) if continuous and approximately normally distributed and median (interquartile range (IQR)) if continuous and not normally distributed. Scores on the PAID, PHQ-4, WHO-5 were treated as binary categorical using clinically relevant cut-off values.

The correlations between the WSE items were examined using cross-tabulation and Pearson’s correlation coefficient (r). Linear regression was used to estimate the unadjusted and adjusted differences in comfort with weight and eating pattern by demographic characteristics and emotional well-being. Statistical analysis was performed using Stata 15.1 (Stata Corp, College Station, TX) and a p-value < 0.05 was considered statistically significant throughout inferential analysis.

## Results

### Participants characteristics

Of 293 young adults aged 15–26 who completed the DPAT, 276 were included (Table 1). Sixteen participants were excluded because they had other types of diabetes one did not complete the WSE items.

**Table 1:** Participant characteristics (demographic, clinical and emotional well-being)

	Median (IQR^†^) or n (%)
*Demographic Characteristics*	
Age (years)	20 (18–22)
*Age group*^‡^	
15–17 years	41 (15%)
18–21 years	153 (55%)
22–26 years	82 (30%)
* Sex*	
Male	130 (47%)
Female	146 (53%)
Body mass index (BMI)^§^ (kg/m^2^)	24.0 (21.6–27.3)
*BMI group*^§^	
Underweight (< 18 kg/m^2^)	14 (6%)
Normal weight (18 to < 25 kg/m^2^)	127 (55%)
Overweight (25 to < 30 kg/m^2^)	54 (23%)
Obese (≥ 30 kg/m^2^)	37 (16%)
*Clinical characteristics*	
Diabetes duration (years)	10 (6.8–14.1)
< 5 years	51 (18%)
≥ 5 to < 10 years	87 (32%)
≥ 10 years	138 (50%)
*Insulin regimen*	
Injections	172 (63%)
Pump	103 (37%)
HbA1c (%) ^¶^	8.5 (7.7–9.8)
HbA1c (mmol/mol)	69 (61–84)
* HbA1c*	
≤ 7%; ≤ 53 mmol/mol	20 (7%)
> 7%; > 53 mmol/mol	255 (93%)
*Emotional well-being*	
Moderate-severe diabetes distress (PAID ≥ 30)	83 (30%)
Presence of anxiety symptoms (PHQ-4 Q1 + Q2 ≥ 3)	71 (26%)
Presence of depressive symptoms (PHQ-4 Q3 + Q4 ≥ 3)	44 (16%)
Likely Depression (WHO-5 × 4 ≤ 28)	39 (14%)

N = 276, Missing data: BMI (n = 3 for continuous BMI + additional n = 41 aged < 18 years for categorical BMI), insulin regimen (n = 1), HbA1c (n = 1)

^†^Interquartile Range

^‡^4 transferred to young adult clinic before age of 16, for 1 transfer to adult clinic was delayed

^§^Restricted to 18 years and older

^¶^HbA1c: glycated haemoglobin. A 7% (53 mmol/mol) HbA1c target was selected as per recommendations from NICE Guideline [45]

The median age was 20 years with similar proportions of males (47%) and females (53%). Median BMI was 24.0 kg/m^2^, median diabetes duration was 10 years and 7% of participants had a HbA1c less than 7%.

Age groups were selected in specific ranges for multiple reasons. Given categorical BMI was not calculated for those aged between 15 and 17, they were allocated a separate group. Creating cohorts for participants aged 18–21 and 22–26 was to achieve an even distribution across the groups.

Nearly one in three participants experienced moderate-severe diabetes distress (PAID scores ≥ 30), 26% presented with anxiety symptoms (PHQ-4 scores ≥ 3 for first two questions) and 16% presented with depressive symptoms (PHQ-scores scores ≥ 3 for last two questions). 14% had likely depression (WHO-5 scores ≤ 28).

### Comfort with current weight, body shape and eating pattern

29% of participants scored 1 or 2 for each item indicating low comfort (Table 2). The percentage who reported low comfort was much higher for females (35–41%) than for males (18.5%-21.5%).

**Table 2:** Comfort with current weight, body shape and eating pattern

Comfort with	N (%) low comfort ^‡^		
	Total	Females	Males
Current weight	82 (29%)	58 (40%)	24 (18.5%)
Body shape	80 (29%)	59 (41%)	21 (16%)
Eating pattern	79 (29%)	51 (35%)	28 (21.5%)

^‡^Scores of 1 or 2, indicating low comfort

Missing data: Comfort with body shape (n = 1)

There was a strong positive correlation between comfort with current weight and comfort with body shape (r = 0.90, *p* < 0.001) with 75% (207/276) of responses being identical for both questions. There was a moderate to strong correlation between comfort with eating pattern and comfort with current weight (r = 0.69, *p* < 0.001) and body shape (r = 0.70, *p* < 0.001). Given the strong correlation between comfort with weight and body shape and the similarity of the concepts, body shape was excluded from the remaining analyses.

### Associations of comfort with current weight and eating pattern with demographic and clinical characteristics and emotional well-being

Females had lower scores than males for comfort with weight (mean [SD] 2.9 [1.3] versus 3.7 [1.1], *p* < 0.001) and eating pattern (3.0 [1.2] versus 3.6 [1.2], *p* < 0.001) (Table 3). Comfort with weight (*p* < 0.001) and eating pattern (*p* = 0.001) was strongly associated with BMI (in participants 18 years or older), with the lowest scores seen in those with BMI ≥ 30 kg/m^2^. Analysis of categorical BMI was restricted to those aged 18 years and older, given that for children and adolescents (up to the age of 17), age and sex are considered when assessing their BMI to account for changes in their body composition as they grow [29]. There was no difference by age group (comfort with weight *p* = 0.39, comfort with eating pattern *p* = 0.38), diabetes duration (*p* = 0.84, 0.27), method of insulin administration (*p* = 0.77, 0.93) or HbA1c (*p* = 0.25, 0.16).

**Table 3:** Comfort with current weight and eating pattern by participant characteristics

		Comfort with			
	n	Current weight		Eating pattern	
		mean (SD^†^)	p-value ^‡^	mean (SD^†^)	p-value ^‡^
*Sex*					
Male	130	3.7 (1.1)	< 0.001	3.6 (1.2)	< 0.001
Female	146	2.9 (1.3)		3.0 (1.2)	
*Age group*					
15–17 years	41	3.5 (1.2)	0.39	3.5 (1.3)	0.38
18–21 years	153	3.2 (1.3)		3.3 (1.3)	
22–26 years	82	3.2 (1.3)		3.2 (1.1)	
*BMI *^*§*^					
Underweight (< 18 kg/m^2^)	14	3.7 (0.9)	< 0.001	3.7 (1.0)	0.001
Normal weight (18 to < 25 kg/m^2^)	127	3.6 (1.2)		3.4 (1.2)	
Overweight (25 to < 30 kg/m^2^)	54	3.0 (1.2) ^*^		3.2 (1.1)	
Obese (≥ 30 kg/m^2^)	37	2.1 (1.1) ^**^		2.6 (1.2) ^**^	
*Diabetes duration (years)*					
< 5	51	3.2 (1.3)	0.84	3.1 (1.3)	0.27
≥ 5 to < 10	87	3.2 (1.3)		3.2 (1.2)	
≥ 10	138	3.3 (1.3)		3.4 (1.2)	
*Insulin administration*					
Injections	172	3.2 (1.3)	0.77	3.3 (1.2)	0.93
Pump	103	3.3 (1.3)		3.3 (1.3)	
*HbA1c (%)*^***¶***^					
≤ 7% (53 mmol/mol)	20	3.6 (1.4)	0.25	3.7 (1.3)	0.16
> 7% (53 mmol/mol)	255	3.2 (1.3)		3.3 (1.2)	
*Moderate-severe diabetes distress (PAID* ≥ *30)*					
No	193	3.5 (1.2)	< 0.001	3.6 (1.2)	< 0.001
Yes	83	2.6 (1.3)		2.6 (1.1)	
*Presence of anxiety symptoms (PHQ-4 Q1* + *Q2* ≥ *3)*					
No	205	3.5 (1.2)	< 0.001	3.5 (1.2)	< 0.001
Yes	71	2.6 (1.3)		2.6 (1.2)	
*Presence of depressive symptoms (PHQ-4 Q3* + *Q4* ≥ *3)*					
No	232	3.3 (1.2)	0.001	3.4 (1.2)	< 0.001
Yes	44	2.7 (1.4)		2.7 (1.2)	
*Likely depression (WHO-5* × *4* ≤ *28)*					
No	237	3.4 (1.2)	< 0.001	3.4 (1.2)	< 0.001
Yes	39	2.5 (1.3)		2.4 (1.3)	

N = 276, Missing data: BMI (n = 3 for continuous BMI + additional n = 41 aged < 18 years for categorical BMI), insulin regimen (n = 1), HbA1c (n = 1)

^†^SD: standard deviation

^‡^Univariable linear regression

^§^Body mass index, restricted to 18 years+

^**¶**^Glycated haemoglobin

*Different to normal weight (*p* < 0.05),

**Different to normal weight (*p* < 0.05) & Different to overweight (*p* < 0.05)

There was no statistically significant difference between underweight and normal weight

Participants with impaired well-being felt less comfortable with their weight and eating pattern than participants reporting optimal well-being (Table 3) (*p* < 0.001 for each association).

Given the strong associations between comfort with current weight and eating pattern with sex and BMI (Table 3), models adjusted for sex and BMI were examined. The adjusted associations were similar to the unadjusted indicating that sex and BMI do not explain the associations between comfort with weight or eating pattern and emotional well-being (Table 4).

**Table 4:** Differences in comfort with current weight or eating pattern by emotional well-being, unadjusted and adjusted for Sex and BMI

	Comfort with current weight	Comfort with eating pattern
	Difference (95% CI)	Difference (95% CI)
*Moderate–Severe diabetes distress (PAID* ≥ *30)*		
Unadjusted†	− 1.0 (− 1.3 to − 0.7)	− 1.0 (− 1.3 to − 0.7)
Adjusted ‡	− 0.8 (− 1.1 to − 0.5)	− 0.8 (− 1.1 to − 0.5)
*Presence of anxiety symptoms (PHQ-4 Q1* + *Q2* ≥ *3)*		
Unadjusted	− 0.9 (− 1.2 to − 0.5)	− 0.9 (− 1.2 to − 0.6)
Adjusted	− 0.7 (− 1.0 to − 0.4)	− 0.7 (− 1.1 to − 0.4)
*Presence of depressive symptoms (PHQ-4 Q3* + *Q4* ≥ *3)*		
Unadjusted	− 0.7 (− 1.1 to − 0.3)	− 0.7 (− 1.1 to − 0.3)
Adjusted	− 0.7 (− 1.0 to − 0.3)	− 0.7 (− 1.1 to − 0.3)
*Likely depression (WHO-5* × *4* ≤ *28)*		
Unadjusted	− 0.8 (− 1.2 to − 0.4)	− 1.0 (− 1.4 to − 0.6)
Adjusted	− 0.7 (− 1.1 to − 0.4)	− 1.0 (− 1.4 to − 0.6)

^†^N = 276 for unadjusted

^‡^N = 273 for adjusted (Continuous BMI missing for n = 3)

## Discussion

This study explored how young adults with T1D perceived comfort with their WSE and the association with demographic, clinical and psychological parameters.

One in three participants reported low comfort with their WSE. The moderate to strong correlations between the three items indicates that the concepts are highly inter-related.

Comparing this to the general population, the Mission Australia’s Youth Survey, included a total of 21,846 young people aged 15–19 years [30]. Body image was among the top three personal concerns for young adults with around three in ten respondents (29%) being extremely or very concerned about body image, which is very comparable to our study findings.

Low comfort with weight and eating pattern was associated with sex; females were more likely to feel less comfortable with their weight and eating pattern, than males. However, these concerns should not be ignored in males, as one in five also experienced low comfort. Literature states that in females a lower body size is socially construed as preferable, whereas for males, body dissatisfaction may come from being thin or short [4, 6, 20, 31].

Young adults older than 18 years of age, with BMI ≥ 30 kg/m^2^ reported lower comfort with their weight and eating pattern compared to those with a BMI indicating normal weight or underweight. Higher BMI is a known predictor of greater body dissatisfaction or risk factor for an ED [32]. This is consistent with the Diabetes MILES Youth study where 62% of female adolescents reported that losing weight was an important goal for them [33]. Whilst not all young people with elevated BMI expressed lower comfort with WSE, our study suggests that in areas with limited resources, people with diabetes who are female and/or have a higher BMI, the treating clinician should further explore the young person’s concerns about weight and body shape. This will inform the most appropriate treatment pathways, such as dietary or psychology support as the next step.

In our study, participants with moderate-severe diabetes distress, presence of anxiety or depressive symptoms were less comfortable with their weight and eating pattern than participants with optimal psychological well-being. This is in keeping with other studies that also confirmed the correlation between severe diabetes distress and DE symptoms [34, 35].

The WSE questions in the DPAT are intended to be quick, easy to understand and answer. They can be used to inform HCP on individuals who may have DE cognitions and allows for early intervention. Low scores (of 1 or 2) prompt a conversation with the clinician. Not all participants scoring low on the WSE are necessarily at risk for DE/ED. Occasionally, the concerns are appropriate, such as simply wanting to “eat healthier” or lose/gain weight for legitimate reasons. This is the reason for referral to the dietitian initially as opposed to the psychologist. The referrals had a high uptake by the young adults, suggesting the WSE items allow identification of young adults who are open to receiving early intervention [21, 36].

Early identification of WSE concerns can identify individuals who may benefit from early interventions to both prevent DE or treat established DE/ED [6, 37–40]. One example of a promising ED prevention program is the Diabetes Body Project. Thirty-five young females with T1D and body image concerns participated group-based ED prevention program [37]. Within 7-days of completing the 6-week program, they reported decreased ED risk factors including body dissatisfaction, internalization of the thin beauty ideal, and general and diabetes-specific ED psychopathology. The benefits of prevention programs have been demonstrated up to 3-months post intervention highlighting the importance of early intervention to prevent progression of ED [40].

Strengths of our study include the large sample size of young adults attending a tertiary-referral centre that services a wide area of metropoliton Queensland. We have previously reported 100% acceptibility and completion of the DPAT questionnaire in our young cohort and near complete data collection [21]. There is minimal missing data apart from categorical BMI, where 41 participants (15%) between the ages of 15–17 were intentionally excluded, given categorical BMI for those under 18 requires age and sex-based cut offs (Tables 1, 3). Those between the ages of 15–17 were included for continuous BMI analysis (Table 4). Another strength of our study is that it provides data for both males and females, demonstrating that some males also reported discomfort with WSE. This inclusivity enhances the robustness of our findings and underscores the utility of our tool in identifying and addressing these concerns across the young adult population.

A limitation is that the WSE items are not validated screening questions and were determined based on literature by consensus in a MDT meeting. While there are other validated tools to screen for DE, such as Diabetes Eating Problem Survey-Revised (DEPS-R) and the modified SCOFF (m-SCOFF) questionnaire, it was decided not to incorporate these into the DPAT for the following reasons [41, 42]. Firstly, the DPAT was intended to be a broad psychosocial screening tool aiming to assess a range of psychosocial concerns. Secondly, adding another tool would elongate the questionnaire and be more burdensome for the young adult to complete. Furthermore, a recent systematic review reported that further research is warranted to evaluate the validity and reliability of currently available tools against the gold standard diagnostic interview [43]. Another limitation is that only biological sex could be obtained from the electronic medical records (EMR). During the years of data collection (2016–2020), the data entered into the EMR was only Male or Female, hence this is the only data that our study can report on. In future studies it would be important to collect self-identified gender and have the appropriate categories available to young people attending the Diabetes Service, to choose from. Existing literature indicates an association between DE and low social determinants of health, such as parental education level, household income, and private health insurance [3]. Although these factors were not examined in our study, they can be evaluated in future research, given that six questions assessing financial concerns are included in the DPAT.

Despite these limitations, the WSE questions reflect concerns people with T1D have related to their weight, body shape and eating pattern which are common in all types of ED. The items have allowed initiation of a conversation with those who may be at risk of DE/ED and allow early referral to allied health staff. Due to the retrospective, cross-sectional design of the study, despite the strong association between the weight and eating concerns with other psychological parameters, we cannot determine whether there is a causal relationship or whether screening for one will infer the other. We can merely comment on the association in this cross-sectional study and suggest that both need to be addressed concurrently in clinical care.

There are opportunities for further research. This finding could inform the fine-tuning of the WSE items in the DPAT. For example, asking a single question regarding comfort with either weight or shape or eating pattern, rather than all three, could be tested in a revised DPAT. It would be important to consider adding an item about self-identified gender and insulin omission when revising the DPAT. It would also be an appropriate step to invite the young person who reports WSE discomfort to complete a DE specific questionnaire following the initial screening procedures which is in line with the recently published guidelines [6]. However, at the time of the DPAT introduction a standard referral pathway to a dietitian and/or a psychologist as needed was established instead. This was because having low WSE comfort, could be a legitimate concern with weight due to an elevated BMI.

The impact of the DPAT referral pathways on the youth with T1D psychological well-being could be further explored. It would also be valuable to undertake future validation studies of the WSE questions as a first-line screening tool for DE/ED. This first analysis has established that the associations of the WSE with demographic variables are in line with existing evidence regarding DEB. For example, the prevalence of positive DEPS‐R was higher among young females compared to males, and in those with increased body mass index values [3, 42, 44]. Hence, the findings from our study, aligning with the literature based on other tools, further validate the comprehensiveness and applicability of our WSE tool in real-world clinical settings.

## Conclusion

In this group of youth with T1D, one in three reported low comfort with their weight, body shape and eating pattern, more so in females and those with a BMI ≥ 30 kg/m^2^. There was a significant association between low comfort with weight and eating pattern and impaired psychological well-being. These results assist in recognising the extent and the profile (demographic/ clinical/ psychological) of the young individuals with these concerns in a real-world Diabetes Service. Using the WSE questions, allows the HCP to start a conversation with these individuals and consider referrals to relevant members of the MDT in a timely, stepwise process.

## Data Availability

The datasets used and/or analysed during the current study are available from the corresponding author on reasonable request.

## Appendix

### Appendix 2: Suggested referral pathways as per the DPAT

Note: All referrals were at the discretion of the treating healthcare professional. The DPAT scores act as a guide.

- Suggested referral to a Credentialled Diabetes Educator: a PAID score ≥ 30 (moderate-severe diabetes distress), or concerns with hypoglycaemia
- Suggested referral to a diabetes psychologist or consideration of initiation of a general practice mental health care plan: A PHQ-4 score ≥ 3 for items 1 + 2 (symptoms of anxiety) or items 3 + 4 (symptoms of depression), or a WHO-5 score ≤ 28 (likely depression)
- Suggested referral to Dietitian: A score of 1 or 2 (suggestive of low comfort) for any of the WSE items

## Abbreviations

DE: Disordered eating
T1D: Type 1 diabetes
ED: Eating disorder
HP: Health professionals
WSE: Weight, body shape and eating pattern
DPAT: Diabetes psychosocial assessment tool
PAID: Problem areas in diabetes
CDE: Credentialled diabetes educator
PHQ-4: Patient health questionnaire-4
WHO-5: World Health Organisation-Five Well-being Index
CES-D: Centre for Epidemiologic Studies Depression Scale
BMI: Body mass index
SD: Standard deviation
IQR: Interquartile range
